# Incorporating anti-infective drugs into peripherally inserted catheters does not reduce infection rates in neonates

**DOI:** 10.3389/fped.2023.1255492

**Published:** 2024-01-05

**Authors:** Julia Koppitz, Rudolf Georg Ascherl, Ulrich Herbert Thome, Ferdinand Pulzer

**Affiliations:** ^1^ Neonatologie, Universitätsklinikum Leipzig, Leipzig, Germany; ^2^ Kinder- und Jugendklinik, Universitätsmedizin Rostock, Rostock, Germany

**Keywords:** neonate, preterm, central line, catheter-related infections, medicated catheters

## Abstract

**Purpose:**

This study assesses whether peripherally inserted central venous catheters (PICC), impregnated with anti-infective drugs, reduce the rate of infections in neonates compared with unimpregnated catheters.

**Methods:**

A retrospective analysis was conducted on electronic patient records of neonates born between August 2014 and May 2020, who had PICCs inserted, either standard (S-PICC) or with anti-infective drugs (A-PICC). Catheter-related bloodstream infections (CRBSI) were diagnosed based on clinical symptoms, laboratory results, and mentioning of infection in the patient record. Data on dwell time, mechanical ventilation, insertion site, maximum C-reactive protein (CRP) concentration, and anti-infective drug use were analyzed.

**Results:**

A total of 223 PICCs were included. The infection rates were A-PICC (18.9%) and S-PICC (12.5%), which were not significantly different (*p* = 0.257). A-PICCs had significantly longer dwell times than S-PICCs (median 372 vs. 219 h, *p* = 0.004). The time to infection was not different between the groups (*p* = 0.3). There were also no significant differences in maximum CRP, insertion site abnormalities, or anti-infective drug use between the groups.

**Conclusion:**

This retrospective study did not find a significant reduction in infection rates by using PICCs containing anti-infective drugs in neonates. Current antibiotic impregnations do not seem to be effective in preventing blood stream infections.

## Introduction

Peripherally inserted central venous catheters (PICC) remain an important tool to ensure adequate parenteral nutrition and application of intravenous medications in small preterm infants for bridging the time until full enteral nutrition has been established (1–5). PICCs are introduced into peripheral veins—most commonly *Vena saphena*, *Vena basilica*, *Vena cephalica*, and the veins of the dorsal hand (1, 2, 6). They are advanced until the tip is located in one of the caval veins (2, 3, 7). Catheter-related blood stream infections (CRBSI) are a common and serious complication (5, 8).

PICCs impregnated with the anti-infective drugs rifampicin and miconazole were designed to reduce CRBSI (9). No recommendation has been made on their routine use in neonates due to a lack of evidence (10). Only the Infusion Nurse Society of the USA suggests using them in high-dependency patients (11). Flemmer et al. spoke at the 42nd conference of the German Society for Neonatology and Pediatric Intensive Care about a reduction in bacteriological complications (12). The multicenter randomized PREVAIL trial did not find a significant difference regarding infection rates (13). Similarly, a monocentric retrospective study at Doha (Qatar) including several catheter types did not find a benefit of anti-infective impregnated catheters regarding culture proven bloodstream infections (14). The following study made use of a large database of patient records accumulated at our university hospital to assess infection rates with PICCs impregnated or non-impregnated with anti-infective drugs in the clinical routine.

## Methods

### Setting

This study was conducted at the University of Leipzig Medical Center, a large tertiary care perinatal center in the German state of Saxony. Due to the retrospective nature of this study, neither treatment nor outcomes were influenced by this investigation. Anti-infective drug-incorporated PICCs (A-PICC, Premistar®, Vygon) were automatically identified from electronic patient records of all patients born between August 2014 and May 2020. This interval was chosen because both types of catheters were inserted during this time. An approximated number of the standard, i.e., drug-free, standard PICCs (S-PICC, Premicath®, Vygon) were randomly selected. PICC that were started during infection by other endovascular devices were excluded as were ambiguously documented PICC. We analyzed all microbiology studies taken during dwell time of the studied PICCs from catheter tips and blood cultures drawn peripherally.

### Definition of CRBSI

Based on the National Health Safety Networks (NHSN) (7) our definition of CRBSI considered three criteria: (i) clinical symptoms, (ii) laboratory results, and (iii) mention of an infection in the patient record (15).

Any PICC was considered infected if two of these three criteria were positive within 7 days (7):

(I) Clinical symptoms were considered positive if at least five of the following eight parameters were present (7, 15, 16): (a) central body temperature >38.5°C, (b) difference between central and peripheral body temperature >2.5°C, (c) tachycardia >180 bpm, (d) bradycardia >6 alarms within 2 h, (e) FiO_2_ increase >20% from baseline, (d) SpO_2_ > 6 alarms within 2 h, (e) pathological alteration of skin color, and (f) prolonged capillary refill time >3 s. Of note, the values for subcriteria (a), (b), (c), and (e) are recorded in our electronic patient records as 15 min averages; they are considered to be positive if the threshold was passed at least once.
(II) Laboratory results were positive if either CRP > 10 mg/L or IL-6 > 200 ng/L.
(III) Documentation was positive if either a nurse or doctor logged an infection in the patient record at that time.

The time of the first positive criterion was regarded as the start of the infection.

Any documented site of infection other than the PICC including other CRBSI led to the exclusion of the infection according to this definition.

### Data acquisition and statistics

Our hospital's Data Integration Center extracted the data from our electronic patient data management system. Additional data were extracted from the hospital information and quality assurance systems. We used the R software environment version 3.6 (17) with the packages *survival* (18), *survminer* (19), and *ggpubr* (20) for time-to-event analyses. If not stated otherwise, discrete variables were compared with Welch's two sample *t*-test or Wilcoxon's rank sum test if normality could not be assumed, categorical variables, with either *χ*² test, or, if the former was inappropriate, Fisher's exact test against the canonical α = 0.05. The results are reported as median [IQR] unless noted otherwise. Data collection and analysis were approved by the institutional review board of the medical faculty of the University of Leipzig (017/20-ek).

## Results

### Sample description

Patient data are listed in Table 1; of note, patients receiving A-PICCs on average had a lower weight (0.90 [0.68, 1.47]) than patients receiving S-PICCs (1.35 [0.87, 2.26], W = 2834.5, *p* < 0.001), and a lower gestational age (A-PICC 27 4/7 wks [25 3/7, 31 2/7] vs. S-PICC 30 3/7 wks [27 2/7, 34 6/7], W = 2878, *p* < 0.001). One patient died in the S-PICC group of fulminant sepsis, 16 days after the last PICC had been removed.

**Table 1:** Patient characteristics by catheter type used (comparisons are *χ*²-test for categorical and *t*-test for discrete variables).

Characteristics	S-PICC (*n* = 112)	A-PICC (*n* = 111)	*p*
Patients	93	88	
Sex Female	41 (44.1%)	33 (37.5%)	0.454
Number of catheters (mean (SD))	1.43 (0.88)	1.50 (0.90)	0.597
Gestational age [days] (median [IQR])^a^	213.00 [191.00, 244.00]	193.00 [177.75, 219.00]	0.001
Gestational age [weeks] (median [IQR])	30 3/7 [27 2/7, 34 6/7]	27 4/7 [25 3/7, 31 2/7]	0.001
Birth weight [kg] (median [IQR])^a^	1.35 [0.87, 2.26]	0.90 [0.68, 1.47]	<0.001
Z-score birth weight (mean (SD))	−0.32 (1.17)	−0.39 (1.57)	0.732
Birth length [cm] (median [IQR])^a^	38.50 [33.00, 44.50]	34.00 [31.00, 40.12]	0.002
Z-score birth length (mean (SD))	−0.64 (1.59)	−0.68 (1.69)	0.886
Head circumference at birth [cm] (median [IQR])^a^	27.50 [23.50, 31.00]	25.00 [22.50, 29.00]	0.005
Z-score head circumference at birth (mean (SD))	−0.45 (3.00)	−0.25 (1.38)	0.570
APGAR-score at 5 min <7	29 (31.2%)	22 (25.0%)	0.448
APGAR-score at 10 min <7	17 (18.3%)	12 (13.6%)	0.517
RDS >2°	16 (17.2%)	32 (36.4%)	0.006
BPD	14 (15.1%)	20 (22.7%)	0.258
IVH >2°	9 (9.7%)	8 (9.1%)	1.000
SIP (+surgery)	5 (5.4%)	14 (15.9%)	0.039
NEC (+surgery)	1 (1.1%)	1 (1.1%)	1.000
CRIB >10	74 (79.6%)	71 (80.7%)	0.999

BPD, bronchopulmonary dysplasia; CRIB, Clinical Risk Index for Babies; IVH, intraventricular hemorrhage; NEC, necrotizing enterocolitis; RDS, respiratory distress syndrome; SIP, spontaneous intestinal perforation.

^a^ Wilcoxon test.

### Primary outcome

**Figure 1:**
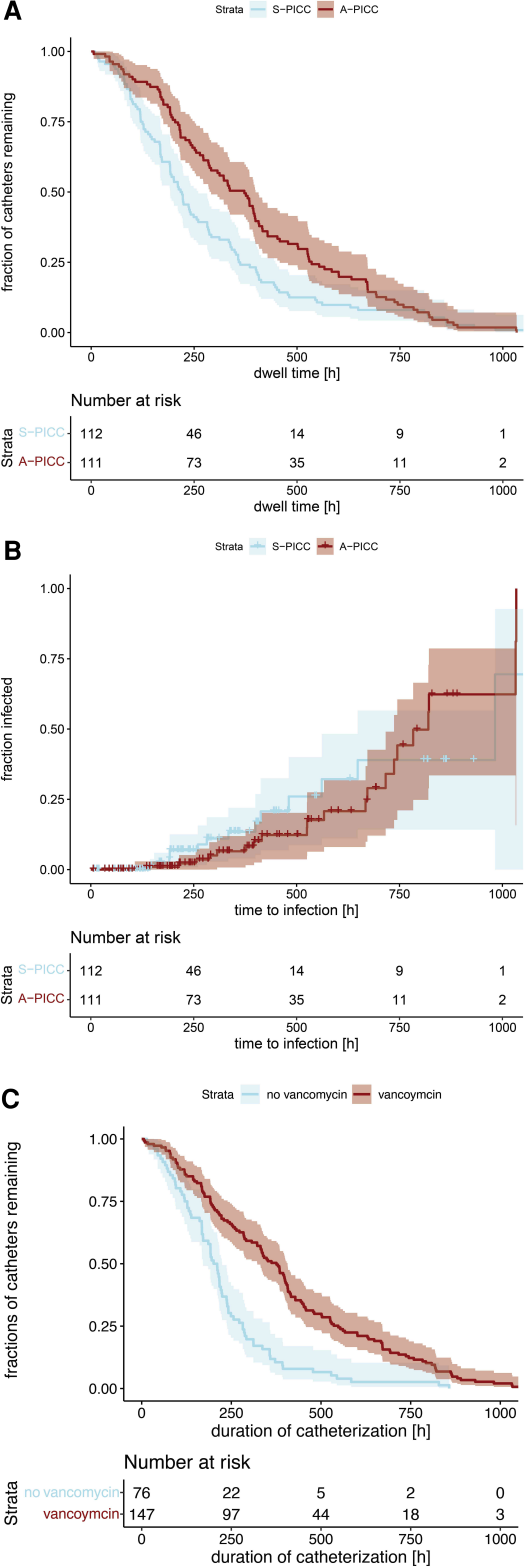
(**A**) Kaplan–Meier curves of catheter dwell time grouped by anti-infective incorporation. (A-PICC median 372 h [95%CI: 291,399] vs. S-PICC 219 h [192,260], *χ*^2^(1) = 8.1, *p* = 0.004). (**B**) Kaplan–Meier curves of time to infection. No difference was demonstrable regarding anti-infective incorporation (*p* = 0.3). (**C**) Kaplan–Meier curves of catheter dwell time grouped by use of vancomycin during dwell time. If vancomycin was used, PICCs had a significantly longer dwell time (with vancomycin 376 h [95%CI: 322,407], without 206 h [171,224], *χ*2(1) = 35.1, *p* < 0.001).

Catheter-related infections were found in 15.7% of the PICCs, but not less often in A-PICCs (18.9%) than in S-PICCs (12.5%) (*χ*^2^(1) = 1.285, *p* = 0.257). Furthermore, A-PICCs had a significantly longer dwell time (Figure 1A) (A-PICC median 372 h [95%CI: 291,399] vs. S-PICC 219 h [192,260], *χ*^2^(1) = 8.1, *p* = 0.004). The time to infection was not different between the two groups (Figure 1B) (*p* = 0.3).

### Catheter ingrowth

To prevent ingrowth, a slight retraction was performed in 31 PICCs during dwell time; there was no difference between the groups (*p* = 0.257) or presence of catheter infection (*p* = 0.287); but PICCs were described as difficult to remove in four cases, all of them had a catheter-related infection (*p* = 0.013).

### Mechanical ventilation

Infants not requiring any ventilator support never met the catheter infection definition (*p* = 0.018). If infants without catheter infection needed mechanical ventilation, it was more often non-invasive than invasive (*χ*^2^(1) = 16.037, *p* < 0.001). Invasive ventilation was needed more often in A-PICC (*χ*^2^(1) = 5.874, *p* = 0.015) and in catheter infections (*χ*^2^(1) = 32.9, *p* < 0.001). The patients with S-PICCs had significantly more often no mechanical ventilation (*χ*^2^(1) = 7.227, *p* = 0.007).

### Insertion site

Abnormalities, such as redness, at the insertion sites were not different between A-PICC and S-PICC (*p* = 0.773). Catheter locations were not different (see Table 2), but infections were more common in catheters inserted at the lower extremities (*χ*^2^(1) = 4.104, *p* = 0.043).

**Table 2:** Insertion site of catheters grouped by infection and anti-infective incorporation. Infection was more common in catheters in the lower extremities (*χ*2(1) = 4.104, *p* = 0.043).

Insertion site	S-PICC (*n* = 112)	A-PICC (*n* = 111)	*p*	No infection (*n* = 188)	Infection (*n* = 35)	*p*
Head	2 (1.8%)	7 (6.3%)	0.102	7 (3.7%)	2 (5.7%)	0.635
Upper extremities	81 (72.3%)	81 (73.0%)	1.000	141 (75.0%)	21 (60.0%)	0.105
Lower extremities	23 (20.5%)	17 (15.3%)	0.400	29 (15.4%)	11 (31.4%)	0.043

### Maximum CRP

Maximum CRP during dwell time was not different between the two groups (*p* = 0.410), but differed, as expected, between infected and uninfected PICCs (infected 74.36 mg/l [25.73, 104.00] vs. uninfected 5.99 mg/l [1.52, 33.48], W = 3766, *p* < 0.001).

### Post-removal

Records were analyzed for a further 48 h after PICC removal. In infected catheters, SpO_2_ alarms were more common (*χ*^2^(1) = 12.757, *p* < 0.001), although notably no abnormalities in body temperature or skin color occurred. The maximum CRP after removal was above reference values, but not different between the two groups (A-PICC 13.59 mg/l [2.74, 35.43] vs. S-PICC 5.63 mg/l [1.85, 13.70], W = 885.5, *p* = 0.089). A higher post-removal CRP was seen in those meeting our infection definition (infected 20.18 mg/l [5.23, 33.10], uninfected 5.95 mg/l [1.57, 17.12], W = 797, *p* = 0.005).

### Microbiology

Peripheral blood cultures were collected more often in cases with infected PICC (infection 48.6% vs. no infection 22.5%, *χ*^2^(1) = 9.007, *p* = 0.003); 7 of 23 cultures in patients with S-PICCs vs. 11 of 36 A-PICCs returned positive, so did 12 of 42 of those with no infection vs. 6 of 17 of the infected; *Klebsiella oxytoca*, *Lactobacillus fermentum, Enterobacter cloacae, Klebsiella pneumoniae, Bacillus subtilis, Bacillus cereus,* and *Serratia marcescens* were found. Skin flora, namely, *Staphylococcus epidermidis, Staphylococcus haemolyticus*, and *Staphylococcus hominis*, were significantly more often detected on infected catheters (*p* = 0.005). Candida antigen studies were not different between the groups.

Anti-infective drugs and resistance: Table 3 sums up aniinfective drug use in relation to the outcomes. In S-PICC, there was significantly more often no anti-infective treatment (A-PICC 5.4% untreated vs. S-PICC 17.9%, *χ*^2^(1) = 7.227, *p* = 0.007). All PICCs meeting the infection definition received anti-infective drugs (infection 100% vs. no infection 86.2%, *p* = 0.018). Five or more anti-infective drugs were used more often in A-PICC (A-PICC 27.0% vs. S-PICC 11.6%, *χ*^2^(1) = 7.555, *p* = 0.006) and in infected PICCs (infection 57.1% vs. no infection: 12.2%, *χ*^2^(1) = 35.403, *p* < 0.001).

**Table 3:** Use of antibiotics by either catheter type and infection and comparisons of dwell times, times to infection, and time to infection or removal with and without anti-infectives.

Anti-infective drugs	Catheter type			Infection definition met			Dwell time			Time to infection			Time to infection or removal		
	S-PICC (*n* = 112)	A-PICC (*n* = 111)	*p*	No infection (*n* = 188)	Infection (*n* = 35)	*p*	Without antibiotic/antifungal [h] (median [95%CI])	With antibiotic/antifungal [h] (median [95%CI])	*p*	Without antibiotic/antifungal [h] (median [95%CI])	With antibiotic/antifungal [h] (median [95%CI])	*p*	Without antibiotic/antifungal [h] (median [95%CI])	With antibiotic/antifungal [h] (median [95%CI])	*p*
Penicillin-derivates	20 (17.9%)	29 (26.1%)	0.184	36 (19.1%)	13 (37.1%)	0.033	255 [223,292]	385 [214,536]	0.02	273 [237,337]	407 [331,671]	0.006	220 [197,249]	198 [123,350]	0.8
Cephalosporins	39 (34.8%]	43 (38.7%)	0.64	63 [33.5%)	19 (54.3%)	0.032	238 [213,286]	382 [287,478]	<0.001	242 [217,312]	407 [331,602]	<0.001	215 [190,240]	237 [190,336]	0.07
Carbapenems	25 (22.3%)	34 (30.6%)	0.210	37 [19.7%)	22 (62.9%)	<0.001	224 [210,264]	444 (397,602]	<0.001	237 [215,284]	602 [444,862]	<0.001	215 [193,240]	249 [107,402]	0.05
Quinolones	2 (1.8%)	3 (2.7%)	0.683	3 (1.6%)	2 (5.7%)	0.176	277 [238,331]	—	0.4	287 [242,348]	—	0.2	216 [193.2,244]	—	0.3
Macrolids	42 (37.5%)	53 (47.7%)	0.158	81 (43.1%)	14 (40.0%)	0.879	233 [192,286]	334 [284,398]	0.05	249 [211,337]	358 [322,404]	0.08	171 [142,233]	283 [216,339]	0.001
Linezolid	2 (1.8%)	1 (0.9%)	1	3 (1.6%)	0 (0.0%)	1	282 [238,332]	—	0.7	292 [246,357]	—	0.6	216 [193,244]	—	0.9
Metronidazol	3 (2.7%)	3 (2.7%)	1	3 (1.6%)	3 (8.6%)	0.051	273 [229,331]	—	0.07	287 [242,352]	—	0.03	216 [193.2,244]	—	0.3
Cotrimoxazol	2 (1.8%)	1 (0.9%)	1	3 (1.6%)	0 (0.0%)	1	272 [237,322]	—	0.2	287 [242,352]	—	0.4	215 [193,241]	—	0.1
Clindamycin	1 (0.9%)	0 (0.0%)	1	0 (0.0%)	1 (2.9%)	0.157	282 [239,332]	—	0.3	291 [246,357]	—	0.6	216 [197,248]	—	<0.001
Gentamycin	22 [19.6%)	28 (25.2%)	0.402	33 (17.6%)	17 (48.6%)	<0.001	251 [222,292]	421 [282,536]	0.001	256 [224,322]	528 [428,862]	<0.001	220 [200.1,249]	168 [98.4,406]	0.1
Vancomycin	64 (57.1%)	83 (74.8%)	0.008	116 (61.7%)	31 (88.6%)	0.004	206 [171,224]	376 [322,407]	<0.001	211 [171,238]	398 [341,447]	<0.001	192 [166,220]	258 [208,334]	<0.001
Antifungals	17 (15.2%)	27 (24.3%)	0.122	24 (12.8%)	20 (57.1%)	<0.001	242 [217,286]	430 (376,601]	<0.001	242 [217,312]	602 [458,890]	<0.001	216 [193.2,248]	212 [84.8,379]	0.2

Penicillin-derivatives (Ampicillin, Ampicillin + Sulbactam, Piperacillin + Tazobactam) were used significantly more often in infections (infection 37.1% vs. no infection 19.1%, *χ*^2^(1) = 4.572, *p* = 0.032) as were cephalosporins (54.3% vs. 33.5%, *χ*^2^(1) = 4.620, *p* = 0.032), gentamicin (48.6% vs. 17.6%, *χ*^2^(1) = 14.587, *p* < 0.001), and antimycotics (57.1% vs. 12.8%, *χ*^2^(1) = 33.941, *p* < 0.001).

Vancomycin was used more often in A-PICC (A-PICC 74.8% vs. S-PICC 57.1%, *χ*^2^(1) = 6.950, *p* = 0.008). It was used in all but four cases of infection. If vancomycin was used, PICCs had a significantly longer dwell time (Figure 1C) (with vancomycin median 376 h [95%CI: 322,407], without 206 h [171,224], *χ*^2^(1) = 35.1, *p* < 0.001).

Only in three A-PICCs bacteria resistant to the incorporated rifampin were found (*p* = 0.049). The resistance to miconazole was not studied.

## Discussion

In this retrospective analysis, infection rates in A-PICC and S-PICC were similar, with a slight trend toward A-PICC. This is in line with the PREVAIL (13) trial and studies by Klemme et al. (21) and Bayoumi et al. (14). None of them detected differences between A-PICC and S-PICC. If the slight trend toward more infections with A-PICC was indeed an effect, the number needed to harm in our population would result in a high value of 15.58. In contrast, catheters incorporating minocycline and rifampicin had been found to have less infections in older children (22).

Flemmer et al. (12) were the only ones to report a reduction in bacteriological complications by A-PICCs in neonates and preterm infants. Unlike their study, bacterial colonization was not tested in every PICC included in our analysis.

The dwell times of PICCs were considerably longer than reported in the literature (our study 12.3 days, other studies 8.2 and approximately 6.5 days) (13, 21). There were no rules prescribing maximum dwell times in our unit. While the removal of infected PICCs was more often complicated, no new methods—as described by Van Mechelen and Mahieu (23)—or surgery were needed.

Positive cultures were not mandatory for defining an infection, because otherwise many infections would be missed owing to the low yield of blood cultures (24). To maintain specificity, we mandated more clinical and laboratory signs to be present during a fixed time interval as suggested by our national German guideline for counting an episode as an infection (15). We feel this is justified as the decision to start anti-infective treatment is in clinical routine often based on clinical criteria and laboratory results like CRP and interleukin 6. Their combination has been ascribed a high positive predictive value (15). Waiting for microbiology results would delay treatment unacceptably. We relinquished from assessing procalcitonin (PCT) since IL-6 and CRP have been shown to be superior (25, 26).

Preterms with lower birth weights are at an increased risk of late-onset sepsis (LOS) (27) and CRBSI (28); longer dwell times have been associated with a rise in sepsis (27) and CRBSI (28, 29) risks. Interestingly, changing central lines after a fixed time is not recommended, because the replacement line has the same infection risk (29).

Catheter tips were not routinely sent for cultures and documentation of catheter removal is often scant. Any discussion on the microbiology from catheter tips must be interpreted after taking into account their unclear role in the diagnosis of CRBSI (30) and the scarcity of studies included in this analysis. Catheter tip cultures were not assigned a role in the definition of infection by their results or in clinical practice. Arguments have been put forward to forgo them altogether (31). Only with a synchronously drawn blood culture might their results be interpretable (30). Our data are hence not suitable for deriving clear statements on colonization. *Staphylococcus epidermidis* was the most common bacterial species identified at the catheter tip with no difference between PICCs considered infected vs. not infected. Such coagulase-negative staphylococci (CNS) have been described as most abundant there (32) and as a relevant cause of LOS (27) and CRBSI (33) in the literature.

Vancomycin is the drug of choice for CNS infection in the preterm; Rodriguez-Guerineau et al. found it especially effective if combined with rifampicin (16). In our department, vancomycin is being used frequently as an anti-infective prophylaxis if catheters are used beyond a certain dwell time. We were unable to distinguish such prophylaxis from actual treatments in our data; it is thus unclear whether prophylaxis actually prevented infection.

We identified multiple limitations in our data. First, the retrospective design: since the decision between A-PICC and S-PICC was made by the physician inserting it, a sum of subjective judgements may have caused A-PICCs being used in children weighing less and having a lower gestational age, possibly because A-PICCs might have been attributed increased safety for longer dwell times, which might have been considered especially advantageous in smaller infants. Since these differences may be confounding variables, some findings in our data may be difficult to interpret.

A-PICCs in our study were inserted in patients weighing significantly less, but were also used longer. The trend toward slightly higher infection rates in the A-PICC group may hence be rather due to the infection risk being generally higher in more immature infants.

Due to low numbers of infection in both groups, estimated power is 25.04%, which is too low to accept the Null Hypothesis, i.e., to demonstrate practical equivalence. A-PICCs and S-PICCs were inserted in patients different in weight and gestational age to which the literature ascribes different CRBSI risks. This lowers the informative value significantly, especially with regard to comparability with other studies. Our infection definition hinges on infection symptoms and laboratory studies, which we feel is representative of clinical practice. The number of infections in our study would have been severely reduced if we had kept to the definition by NHSN demanding a positive blood culture, because our department orders few microbiological studies. It seems unlikely that in our case more microbiological studies would have helped increase the number of infections since only 4%–12% return positive (34–37). Missing a large number of infections, however, would also not improve the conclusions. Of note, there is a high variability in peripheral venous blood culture positive rates: Klemme et al. had no positive blood cultures, whereas the PREVAIL trial reported 61.3%. Limitation of the analysis to catheter dwell time (plus 48 h) and exclusion if any other site of infection had been documented was further reducing the number of infections. Despite all of these shortcomings, we feel that our definition is very close to what is happening in the clinical routine, where catheter infection is most often diagnosed by ruling out other explanations, and antibiotic treatment is started long before microbiology results are available.

If our observations were used to estimate the sample size of a trial with an ɑ = 0.05 and a power of 90%, about 700 PICCs would be needed in both arms.

Finally, because of the few infections in our study, a clear benefit from repeatedly retracting longer-dwelling PICCs was not discernible.

In spite of these limitations, this manuscript is a description of the current use and complications of PICCs in our department with an infection definition that resembles routine rather than an artificial study environment.

## Conclusion

In line with other studies on this topic, we could find no clear advantage regarding infections by incorporating anti-infective drugs into peripherally inserted central catheters.

If one were still to stipulate more evidence, for deciding whether to use A-PICCs, a larger trial would be required.

## Data Availability

The raw data supporting the conclusions of this article will be made available by the authors, without undue reservation.
